# Personality, Resilience, and Calling in Students Undertaking a Medical Degree Across Two Continents: Disparate Pathways to the Healing Profession

**DOI:** 10.31486/toj.20.0029

**Published:** 2021

**Authors:** David Galarneau, Leonardo Seoane, Diann S. Eley

**Affiliations:** ^1^ Department of Psychiatry, Ochsner Clinic Foundation, New Orleans, LA; ^2^ The University of Queensland Faculty of Medicine, Ochsner Clinical School, New Orleans, LA; ^3^ Department of Pulmonary and Critical Care, Ochsner Clinic Foundation, New Orleans, LA; ^4^ Louisiana State University Health Sciences Center–Shreveport, Shreveport, LA; ^5^ Office of Medical Education, Faculty of Medicine, The University of Queensland, Brisbane, Queensland, Australia

**Keywords:** *Education–medical*, *international medical students*, *personality*, *resilience–psychological*, *students–medical*

## Abstract

**Background:** An educational partnership between The University of Queensland (UQ) in Australia and Ochsner Health in the United States developed the UQ-Ochsner medical program that trains American citizens to practice medicine in the United States. This program provides the opportunity to explore and compare the personal characteristics of UQ-Ochsner students with their domestic (Australian citizen) and international classmates not enrolled in the Ochsner program. Findings may offer some insights into the types of students who choose to study medicine across multiple countries.

**Methods:** We used a quantitative cross-sectional design for our study. A first-year cohort of domestic, international, and UQ-Ochsner students completed a survey comprising demographic questions and measures of temperament and character personality, resilience, and calling to medicine. Univariate statistics were used to compare groups for all variables.

**Results:** The whole sample response rate was 72.1% (375/520). Domestic students represented the greatest proportion of students <25 years, and UQ-Ochsner students represented the greatest proportion of students of ≥26 years. Gender did not differ significantly between groups. The majority first degree for all students was science, although 23% of UQ-Ochsner students reported arts/law/humanities degrees. In comparison to all other students (domestic and international combined), UQ-Ochsner students scored significantly lower in levels of harm avoidance (*P*=0.039) and higher in self-directedness and self-transcendence, resilience, and calling with medium to strong effect sizes (*d*>0.3).

**Conclusion:** UQ-Ochsner students have a personality profile similar to their classmates but with levels of certain traits—higher self-directedness and lower harm avoidance—that in combination contribute to higher resilience and a strong sense of calling to medicine. Being slightly older may allow for more development of self-directedness, but low harm avoidance suggests an innate degree of confidence in and acceptance of risk to achieving goals.

## INTRODUCTION

In 2009, The University of Queensland (UQ) in Brisbane, Australia, and Ochsner Health in New Orleans, Louisiana, USA, formed a unique partnership to provide an innovative international medical education experience across 2 continents. The UQ-Ochsner medical program offers a fully accredited 4-year MD degree whereby eligible American citizens or permanent residents undertake the preclinical phase—years 1 and 2—at UQ in Brisbane and return to the United States for the clinical phase—years 3 and 4—in the Ochsner Clinical School at Ochsner Health. A major feature of the UQ-Ochsner medical program is that American medical students are prepared to pass the United States Medical Licensing Examinations, enter the National Residency Matching Program, and practice medicine in the United States. UQ-Ochsner students have the unique opportunity to study in and experience 2 disparate health care systems at 2 highly ranked institutions. While most US medical schools provide some opportunity for exposure to international training, the students of the UQ-Ochsner medical program are educated on 2 continents and receive half of their medical education in Australia.

International students are not new to Australian medical schools and often comprise up to 40% of cohorts in some schools.^1^ In the increasingly globalized world, complexities of distance and communication are less of an impediment to an international education. While university students may choose an international education experience for many reasons, a common reason among students pursuing medicine is to gain an international perspective on health care congruent with the rapid spread of global health burdens.^2^

A shared mission of the UQ-Ochsner partnership is to offer a strong international element to the UQ medical program and encourage students with an interest in global health to benefit from a varied international peer group. The UQ medical program has a large annual intake of approximately 480 to 520 students. The majority are domestic Australians (60%), with 20% traditional UQ-international students (non-Ochsner), principally from Canada and Asia, and 20% UQ-Ochsner students.

### Why Students Choose to Study Abroad

Tertiary education can present a range of academic and personal stressors, and international students often face particular difficulties. Personally, they have moved away from family and friends, often for the first time, and must navigate new cultural and social norms, as well as a new education system. Academically, they may be under more pressure to succeed because of the additional cost of their education, including living expenses and travel, and may be mindful of sacrifices made on their behalf by family and friends.^3,4^ Medicine is universally recognized as a challenging degree for even the brightest students, regardless of origin. The literature is clear about the increased levels of stress among medical doctors and students in comparison to the general population. International medical students are particularly vulnerable to potential stressors.^5,6^ In a systematic review examining factors that influence decisions by health care and medical students to study overseas, prior knowledge of and sufficient details about the international program were most important, along with an interest in other countries and cultures, and strong motivation from family or academic staff to travel and study abroad.^2^

Global collaborations between overseas hospitals or medical centers and US medical schools are not new. Ben-Gurion University of the Negev Faculty of Health Sciences in Israel collaborated with Columbia University Medical Center in New York City in 1998. Other collaborations include Weill Cornell Medicine and Qatar and a long-established partnership between Duke University and the National University of Singapore.

The UQ-Ochsner medical program has several unique features that distinguish it from other international medical programs. The most distinctive is that American citizens are trained in Australia to practice medicine in the United States. The program integrates the medical degree across 2 continents to enhance students’ global and cross-cultural perspective and apply it to their careers as international medical graduates (IMGs) in the United States.

Traditionally, a stigma is associated with being an IMG, and IMGs typically find success in the US residency match to be difficult. Cognisant of this potential difficulty, UQ-Ochsner students are motivated to seek additional achievement areas to counteract any possible stigma. Research is an area in which UQ-Ochsner students have excelled. The proportion of UQ-Ochsner students who are research active, undertaking projects, publishing their work, and/or pursuing an MD-PhD or MD-MSc degree, exceeds the proportion of domestic Australian and traditional UQ-international (non-Ochsner) classmates involved in these activities. Evidence of the UQ-Ochsner students’ motivation to succeed is demonstrated in the residency match results: they match at a rate equivalent to students of US medical schools despite being classified as IMGs.^7^

A practical consideration of the program is the distance between the United States and Australia. UQ-Ochsner students could not get farther away from family and friends, and while staying connected electronically is routine, when personal or urgent issues arise, the distance can be a time and financial burden that may cause distress. These concerns warrant a good understanding of the personal characteristics of students who choose the UQ-Ochsner medical program. This study focused on comparing the levels of personality traits, resilience, and calling (career calling to become a doctor) between UQ-Ochsner students and their domestic Australian and traditional UQ-international (non-Ochsner) classmates.

### Temperament and Character Personality

Personality represents everyone's unique set of emotions, behaviors, and characteristics underpinned by biological factors and environmental dynamics. Medical education has a long history of investigating personality and its influences on a myriad of variables relevant to medical training. Several studies have noted the importance of considering personality in the assessment of noncognitive characteristics for student selection.^8-11^ Certain traits, such as conscientiousness, appear to be consistently associated with success and positive outcomes in medical student academic achievements,^11^ ability to cope with stress,^12,13^ and clinical competence.^14,15^ However, every individual's personality is made up of a combination of all traits. Looking at single traits may misrepresent an individual's true personality.^16,17^ That combination, or profile of traits, contributes to every person's unique personality and influences the person's attitudes and behaviors in the situational context.

Using the theoretical framework based on the Cloninger psychobiological model, personality is comprised of 2 dimensions—temperament and character.^18^ The 7-factor model comprises 4 temperament traits and 3 character traits assessed by the Temperament and Character Inventory (TCIR-140).^19^ Temperament represents our emotional core; it is stable, moderately heritable, and biologically determined. Temperament traits are novelty seeking (curiosity reflected as bias in response to novelty and challenge), harm avoidance (anxiety reflected in the capacity to accept uncertainty and risk), reward dependence (social attachment reflected as dependence and a need to please), and persistence (behavior despite frustration reflected in perseverance, diligence, and overachievement). Character refers to acquired traits and may be developed and influenced by life experience. Character traits are self-directedness (the dichotomy of responsible vs blaming reflected as conscientious and goal oriented), cooperativeness (the dichotomy of empathic vs insensitive reflected as tolerant and constructive), and self-transcendence (the dichotomy of idealistic vs practical reflected as how one conceives themselves in relation to the wider world). The model further proposes a link between certain temperament traits and specific neurotransmitters (eg, between novelty seeking and dopamine, harm avoidance and serotonin, and reward dependence and norepinephrine).^20^ Character traits are said to be related to insight learning and shaped by temperament, social influences, and environmental factors.^21^

### Resilience

Resilience is a dynamic process that manifests itself in response to life circumstances and one's personality. Resilience as a construct is an indicator of a psychologically mature personality. Several studies, using various measures, have shown that someone who is psychologically mature demonstrates a temperament with low levels of harm avoidance and high levels of persistence, strengthened by high levels of self-directedness and cooperativeness.^22-28^ Therefore, psychological maturity, in its complete sense, is a strong predictor of resilience and the ability to cope with and bounce back from adversity.^29,30^

### Calling

A variety of literature is available on calling, from spiritual connotations to life meaning, life satisfaction, well-being, and motivation.^31,32^ Calling is most often associated with career and vocational development, defined as a transcendent summons to a meaningful career that serves others.^33^ The notion of calling has often been associated with a medical career.^31,34,35^ Differences in levels of calling across health profession students in medicine, pharmacy, and dentistry showed that a significantly higher number of students in medicine acknowledged a calling compared to students of other health professions.^36^ Calling is also associated with purpose and as such may influence goal setting and motivation.^37^ Studies focused on students have found that those with a strong calling are more motivated to invest the effort necessary to reach their goals.^38^ Medical students with high levels of calling are more likely than other medical students to view their life as meaningful. Calling has a strong association with overall psychological health and life satisfaction^31^ and has been linked to self-efficacy and levels of commitment in medical students.^35^

### Objectives and Aims

The introduction of the UQ-Ochsner program provided a natural experiment to compare medical students undertaking the same degree from different educational and cultural perspectives. This study investigated personal characteristics of one cohort of students in the UQ-Ochsner medical program as a means of understanding differences between them and their classmates. Therefore, comparisons between UQ-Ochsner students and the combined domestic Australian and traditional UQ-international (non-Ochsner) students were made, as well as comparisons among all 3 groups. This information may be helpful in recruiting future and counseling current UQ-Ochsner students.

## METHODS

### Study Design

The study design was quantitative cross-sectional using a self-report questionnaire. The UQ Human Research Ethics Committee provided ethical clearance (#2017001468). All participants gave consent on the questionnaire.

### Participants and Setting

Data were collected in 2016. All first-year students in the 4-year MD degree program were invited to participate by completing an online questionnaire via a SurveyMonkey web link during a regularly scheduled activity in orientation week. The survey and its purpose were explained prior to its administration. Students were categorized into 3 groups: Australians as domestic, traditional (onshore) internationals as international, and UQ-Ochsner.

### Measures

The questionnaire included basic sociodemographic questions: age group, gender, first degree obtained, and student group. Personality was measured by the 140-item version of the TCIR-140^19^ using a 5-point Likert scale from 1 (“absolutely false”) to 5 (“absolutely true”). As described and defined earlier, the 4 temperament traits assessed by the TCIR-140 are novelty seeking, harm avoidance, reward dependence, and persistence, and the 3 character traits assessed by the TCIR-140 are self-directedness, cooperativeness, and self-transcendence. Each trait is multifaceted. High and low descriptors are summarized in Table 1.

**Table 1.** Temperament and Character Trait Descriptors^a^

Temperament Traits	Represents	Low Scores	to	High Scores
Novelty seeking	Exploratory activity in response to novelty	Orderly, reflective, tolerant, reserved	↔	Exploratory, curious, seeks challenge
Harm avoidance	Worry in anticipation of problems	Confident, accepting of uncertainty and risk	↔	Worrying, anxious, unable to accept risk
Reward dependence	Dependence on approval of others	Not influenced by others, objective, insensitive	↔	Needs to please, warm, attached
Persistence	Industriousness of behaviour despite obstacles	Quitting, underachiever, erratic, unambitious	↔	Ambitious, diligent, perfectionist
**Character Traits**	**Represents**	**Low Scores**	**to**	**High Scores**
Self-directedness	Responsibility, goal oriented, and self-confidence	Blaming, ineffective, unreliable	↔	Conscientious, reliable, self-accepted
Cooperativeness	Tolerance, cooperativeness, and empathy	Intolerant, unhelpful, opportunistic, critical	↔	Tolerant, agreeable, constructive, empathic
Self-transcendence	View of self in relation to the universe as a whole	Impatient, proud, materialistic, practical	↔	Patient, humble, spiritual, creative

^a^Adapted from Eley et al, 2017^47^

The Brief Resilience Scale^39,40^ is a self-report measure of a person's ability to respond to adversity. The 14-item version uses a 7-point Likert scale from 1 (“strongly disagree”) to 7 (“strongly agree”). The scale reflects 5 core characteristics of resilience: perseverance, equanimity, meaningfulness, self-reliance, and existential aloneness. The single composite score ranges from 14 to 98, with higher scores indicating higher levels of resilience. Low scores are <65, moderate scores are 65-81, and high scores are >81.

The degree to which students viewed their current career as a calling was assessed by the Brief Calling Scale.^41^ Two items from this 4-item scale were included: “I have a calling to particular kind of work” and “I have a good understanding of my calling as it applies to my career.” A 5-point Likert-type response scale from 1 (“not at all true of me”) to 5 (“totally true of me”) was used. Higher scores indicate higher levels of calling. Scores range from 2-10.

### Analyses

The internal consistency (Cronbach alpha) of the TCIR-140 ranged from 0.84 to 0.88 for the character traits and from 0.76 to 0.89 for the temperament traits. The Brief Resilience Scale alpha was 0.89, and the Brief Calling Scale, assessed by Pearson correlations, was 0.89. We used chi-square tests to examine proportions in the demographic variables, and we used *t* tests and 2-way analysis of variance with Bonferroni post-hoc pairwise comparisons to examine differences between traits by group, and by gender and age. The relationship between measures of temperament and character dimensions, resilience, and calling was investigated by Pearson product-moment correlation coefficients (2-tailed) controlling for age and gender. All tests used α=0.05 with an accompanying 95% CI and were analyzed using SPSS statistical software, version 25 (IBM Corp).

## RESULTS

### Participant Response Rate

The overall sample response rate was 72.1% (375/520). The responses of the individual groups were as follows: domestic 82% (230/279), international 58% (70/120), and UQ-Ochsner 62% (75/121).

### Sample Description

Table 2 shows the demographics of the whole sample and by group. The distribution by gender was not different. The median age of the sample was 23 years with a range of 22 years (19-41 years). Age was divided into <25 years and ≥26 years. Age was significantly different between the groups (*P*=0.001), with domestic students representing the greatest proportion of students <25 years and UQ-Ochsner students representing the greatest proportion of students ≥26 years. More than 60% of UQ-Ochsner students reported science as their first degree, and 22% reported degrees in the arts/law/humanities. More than 80% of all other students had science degrees.

**Table 2.** Demographics by Student Group and Overall

Variable	Domestic Students n (%)	UQ-Ochsner Students n (%)	International Students n (%)	Total n (%)	*P* Value
Gender					
Male	150 (65)	43 (57)	35 (50)	228 (61)	
Female	80 (35)	32 (43)	35 (50)	147 (39)	
Total	230	75	70	375	NS
Age group					
<25 years	184 (82)	17 (23)	24 (37)	225 (62)	
≥26 years	41 (18)	58 (77)	41 (63)	140 (38)	0.001
Total	225	75	65	365	
Marital status					
Partnered	31 (14)	12 (16)	8 (11)	51 (14)	
Single	198 (86)	63 (84)	62 (89)	323 (86)	
Total	229	75	70	374	NS
First degree					
Science	189 (83)	48 (64)	58 (83)	295 (79)	0.001
Health science	14 (6)	7 (9)	6 (9)	27 (7)	
Arts/law/humanities	8 (3)	17 (23)	5 (7)	30 (8)	
Engineering/math	18 (8)	3 (4)	1 (1)	22 (6)	
Total	229	75	70	374	

Notes: Pearson chi-square comparisons were made across groups.

NS, not significant; UQ, University of Queensland.

### Personality Traits by Age and Gender of the Whole Sample

Students in the older age group had significantly higher levels of calling (*t*=−2.246; 258; *P*=0.02), novelty seeking (*t*=−2.511; 364; *P*=0.01), persistence (*t*=−2.397; 364; *P*=0.01), self-directedness (*t*=−2.066; 364; *P*=0.04), and self-transcendence (*t*=−2.546; 364; *P*=0.01), and lower levels of harm avoidance (*t*=−2.128; 364; *P*=0.03) than students in the younger age group. Resilience and cooperativeness did not differ by age.

Female students had significantly higher levels of all traits compared to male students except for novelty seeking and self-transcendence, which were not significantly different between groups. The *t* statistics for female vs male traits levels are as follows: calling (*t*=−2.559; 368; *P*=0.01), resilience (*t*=−2.540; 368; *P*=0.01), harm avoidance (*t*=−2.336; 374; *P*=0.02), reward dependence (*t*=−4.224; 374; *P*=0.001), persistence (*t*=−2.279; 374; *P*=0.02), self-directedness (*t*=−3.499; 374; *P*=0.001), cooperativeness (*t*=−4.445; 374; *P*=0.001), and self-transcendence (*t*=−1.617; 374; *P*=0.01).

### Comparison of All Three Groups

Table 3 compares the means and SDs of all traits by all groups. Bonferroni post-hoc pairwise comparisons showed that levels of harm avoidance were significantly lowest in UQ-Ochsner students (*P*=0.039) and levels of self-directedness and self-transcendence were highest in UQ-Ochsner students compared to the other groups. Levels of resilience (*P*=0.004) and calling (*P*=0.001) were significantly lower in domestic students compared to both UQ-Ochsner students and international students.

**Table 3.** Analysis of Variance Comparing the Means and Standard Deviations of Personality Traits by Student Group

Trait	Student Group	n	Mean ± SD	95% CI for Mean	*P* Value
Novelty seeking^a^	Domestic	230	2.70 ± 0.43	2.64-2.75	
	UQ-Ochsner	75	2.82 ± 0.42	2.72-2.91	
	International	70	2.70 ± 0.40	2.60-2.79	
	Total	375	2.72 ± 0.42	2.68-2.76	
Harm avoidance^a^	Domestic	230	2.79 ± 0.71	2.70-2.88	
	UQ-Ochsner	75	2.56 ± 0.66	2.41-2.71	0.039
	International	70	2.74 ± 0.65	2.59-2.90	
	Total	375	2.74 ± 0.69	2.67-2.81	
Reward dependence^a^	Domestic	230	3.39 ± 0.53	3.33-3.46	
	UQ-Ochsner	75	3.45 ± 0.60	3.31-3.59	
	International	70	3.39 ± 0.46	3.28-3.50	
	Total	375	3.40 ± 0.53	3.35-3.46	
Persistence^a^	Domestic	230	3.79 ± 0.56	3.72-3.86	
	UQ-Ochsner	75	3.91 ± 0.47	3.80-4.02	
	International	70	3.88 ± 0.51	3.76-4.01	
	Total	375	3.83 ± 0.53	3.78-3.88	
Self-directedness^a^	Domestic	230	3.62 ± 0.59	3.55-3.70	
	UQ-Ochsner	75	3.85 ± 0.55	3.73-3.98	0.005
	International	70	3.78 ± 0.54	3.65-3.90	
	Total	375	3.70 ± 0.58	3.64-3.76	
Cooperativeness^a^	Domestic	230	4.03 ± 0.44	3.97-4.08	
	UQ-Ochsner	75	4.07 ± 0.42	3.98-4.17	
	International	70	4.12 ± 0.43	4.02-4.22	
	Total	375	4.05 ± 0.43	4.01-4.10	
Self-transcendence^a^	Domestic	230	2.59 ± 0.62	2.51-2.67	
	UQ-Ochsner	75	2.95 ± 0.65	2.80-3.10	0.001
	International	70	2.85 ± 0.58	2.71-2.99	
	Total	375	2.71 ± 0.64	2.65-2.78	
Resilience^b^	Domestic	226	80.76 ± 10.39	79.40-82.12	0.004
	UQ-Ochsner	74	84.99 ± 8.60	82.99-86.98	
	International	69	83.06 ± 9.68	80.74-85.39	
	Total	369	82.04 ± 10.04	81.01-83.07	
Calling^c^	Domestic	226	7.16 ± 2.10	6.88-7.43	
	UQ-Ochsner	74	8.24 ± 1.71	7.85-8.64	0.001
	International	69	8.52 ± 1.47	8.17-8.88	
	Total	369	7.63 ± 2.01	7.43-7.84	

Note: Only *P* values <0.05 are reported.

^a^Temperament and Character Inventory means are calculated based on a Likert scale of 1 to 5: 1.00-1.50=very low, 1.51-2.50=low, 2.51-3.50=average, 3.51-4.50=high, and 4.51-5.00=very high.

^b^Resilience scores range from 14-98. Low scores are <65, moderate scores are 65-81, and high scores are >81.

^c^Calling scores range from 2-10. Higher scores equate to a higher levels of calling.

UQ, The University of Queensland.

### Comparison of UQ-Ochsner With All Other Medical Students

Table 4 shows the comparison between the means for each trait for the UQ-Ochsner cohort vs the combined scores of all other UQ medical students (domestic and international). In comparison to all other students, UQ-Ochsner students scored significantly lower in harm avoidance, and higher in novelty seeking, self-directedness, self-transcendence, resilience, and calling. The effect size (Cohen *d*) of the significance of novelty seeking is weak and should be interpreted with caution. However, the effect sizes measured for harm avoidance, self-directedness, self-transcendence, resilience, and calling are medium to strong (*d*>0.3).

**Table 4.** Comparison of Personality Trait Means ± SDs for The University of Queensland (UQ)-Ochsner Students and All Others (Combined Scores of Domestic and International Students)

Trait	Student Group	n	Mean ± SD	95% CI for Mean	*P* value	Cohen *d*
Novelty seeking^a^	UQ-Ochsner	75	2.82 ± 0.42	0.01 to 0.23	0.026	0.29
	All others	300	2.70 ± 0.42			
Harm avoidance^a^	UQ-Ochsner	75	2.56 ± 0.66	−0.40 to −0.05	0.011	0.33
	All others	300	2.78 ± 0.69			
Reward dependence^a^	UQ-Ochsner	75	3.45 ± 0.60	−0.08 to 0.19	0.414	0.11
	All others	300	3.39 ± 0.51			
Persistence^a^	UQ-Ochsner	75	3.91 ± 0.47	−0.04 to 0.23	0.159	0.19
	All others	300	3.81 ± 0.55			
Self-directedness^a^	UQ-Ochsner	75	3.85 ± 0.55	0.05 to 0.34	0.008	0.34
	All others	300	3.66 ± 0.58			
Cooperativeness^a^	UQ-Ochsner	75	4.07 ± 0.42	−0.09 to 0.13	0.667	0.04
	All others	300	4.05 ± 0.44			
Self-transcendence^a^	UQ-Ochsner	75	2.95 ± 0.65	0.14 to 0.45	0.001	0.46
	All others	300	2.65 ± 0.62			
Resilience^b^	UQ-Ochsner	74	84.99 ± 8.60	1.19 to 6.28	0.004	0.39
	All others	296	81.25 ± 10.27			
Calling^c^	UQ-Ochsner	74	8.24 ± 1.71	0.26 to 1.27	0.003	0.41
	All others	296	7.48 ± 2.05			

Note: Cohen *d* values > 0.3 are considered moderate to strong.

^a^Temperament and Character Inventory means are calculated based on a Likert scale of 1 to 5: 1.00-1.50=very low, 1.51-2.50=low, 2.51-3.50=average, 3.51-4.50=high, and 4.51-5.00=very high.

^b^Resilience scores range from 14-98. Low scores are <65, moderate scores are 65-81, and high scores are >81.

^c^Calling scores range from 2-10. Higher scores equate to a higher levels of calling.

### Relationships Between Traits

Relationships among the personality traits showed several strong (*r*>0.4) significant correlations (*P*<0.01; 2-tailed). Resilience was positively correlated with calling (*r*=0.437), persistence (*r*=0.591), and self-directedness (*r*=0.659) and negatively correlated with harm avoidance (*r*=−0.594). Calling was positively related to persistence (*r*=0.360) and self-directedness (*r*=0.359).

## DISCUSSION

In this study, we investigated personality, resilience, and calling in a cohort of UQ-Ochsner students undertaking a medical degree across 2 continents, North America and Australia. The objective was to compare UQ-Ochsner students and their domestic and international classmates. The study goal was to improve our understanding of students who choose to enroll in the UQ-Ochsner medical program, with the aim of enhancing future recruitment and current counseling of students.

This study has some limitations including the inability to generalize the findings for reasons common to cross-sectional, largely observational studies. The sample came from one cohort of medical students and may not be representative of the larger medical student population. Although beyond the scope of this study, the addition of data from a traditional US medical school would enhance the findings. The data collected were self-reported, and students who were or were not interested in and attentive to the subject matter may have had biased responses.

### Describing the Sample

The combined cohort of UQ-Ochsner, domestic, and international students differed little in demographics except for age and previous degree. More UQ-Ochsner students entered medicine after age 25 years and with an arts/law/humanities degree than domestic or international students. A 2019 study showed that medical students with degrees in humanities or social science perform significantly better in interpersonal skills and communication compared to medical students with science degrees.^42^ Shacklady et al found that students entering medicine just a few years older than age 21 years experienced a better transition from preclinical to clinical learning,^43^ suggesting that life experience and social factors are influential at this age.

Age is also a consideration in terms of personality development and is evident in our data. Ages in our study range from 19-41 (median 23 years), meaning the majority are considered in the young adult stage. This stage is important in cognitive and emotional development, as the prefrontal cortex, which is essential in decision-making capacity, typically reaches maturity around the age of 26 years. Therefore, any differences in temperament and character by age are worthy of interest in terms of personality development.^44^

Looking at the personality trait levels of the whole sample, trends similar to those found in previous studies of medical students are evident.^45-50^ Overall, this sample of students portrays what would be considered a mature personality common in individuals with high intellect and motivation. The mature personality profile is defined primarily by 2 temperament traits and 2 character traits. The temperament traits are average to low levels of harm avoidance (indicating a low tendency toward anxiety) and high levels of persistence (indicating industriousness and perseverance). High levels of the character traits self-directedness (reflecting responsibility and conscientiousness) and cooperativeness (representing agreeableness and empathy) balance the temperament traits to form a personality profile that is highly predictive of well-being and coping. Studies consistently describe medical students, doctors, and other health professionals by this mature profile that has been referred to as resilient.^22,23^

Congruent with other studies, temperament and character traits are associated with certain demographics, particularly age and gender.^23,45-50^ Older students in our sample were lower in harm avoidance and higher in self-directedness, persistence, and self-transcendence than younger students. Older students were also higher in calling, a finding that may indicate a more developed understanding of the purpose of their medical training. Differences in trait levels by gender are widely reported, and our study concurs. Females were significantly higher than males in most traits, including harm avoidance, which suggests a more cautious nature, yet were highly self-directed, persistent, and cooperative in addition to having higher levels of resilience and calling to medicine.

### Comparing UQ-Ochsner Students With Their Classmates

Comparisons of the UQ-Ochsner students with their classmates revealed several differences. In the 3-way comparison shown in Table 3, post hoc tests showed that domestic students were different from both international groups. This finding led us to investigate whether the UQ-Ochsner students were different from their combined classmates (Table 4). The same differences remained but with the addition of a higher mean for novelty seeking in the UQ-Ochsner students. The remaining discussion focuses on this analysis because as we have mentioned, the nature of the UQ-Ochsner program is unique to that experienced by their domestic and international classmates. In particular, the constructs of calling and resilience are germane to this group, as is the higher level of novelty seeking, as possible explanations for entering the UQ-Ochsner program.

Compared to their classmates, UQ-Ochsner students were significantly higher in levels of resilience and calling, with strong effect sizes. This finding aligns with their profile of lower harm avoidance and higher persistence and self-directedness, which has implications for explaining their higher level of resilience.^22^ We found a strong negative correlation between resilience and harm avoidance. Harm avoidance represents a heritable bias toward anxiety and pessimism.^21-23^ Therefore, students low in harm avoidance tend to be more confident and accepting of a degree of risk. On the other hand, high persistence naturally aligns with an inherent tendency to be resilient and bounce back from adversity. However, high persistence is often indicative of detrimental perfectionistic tendencies. High-achieving individuals often portray a degree of perfectionism that is frequently self-defeating and leads to heightened levels of anxiety and burnout.^16,17^ Nevertheless, when combined with high levels of self-directedness, cooperativeness, and low harm avoidance, a synergistic relationship is formed that is conducive to high resilience. The resilient personality illustrates the importance of considering combinations (profiles) of traits, not individual traits.^14,17^

Our data showed a strong correlation between resilience and calling, with UQ-Ochsner students significantly higher in both when compared to their classmates. While UQ-Ochsner students are studying for the same medical degree as their domestic and international classmates, they must overcome several unique challenges to enter the medical profession in the United States. It makes sense that their personalities are conducive to being more resilient along with a purpose to achieve a strong calling. In other words, regardless of the effort involved, a high calling to that effort is likely to complement the resilience to accomplish its pursuit. A study showed that residents with a higher calling to medicine were higher in levels of resilience and lower in symptoms of burnout, stress, and anxiety compared to residents with a lower level of calling to medicine.^51^ Therefore, calling may not only engender a more positive outlook on career development but also serve as a protective factor for mental well-being.

## CONCLUSION

The data show that this cohort of UQ-Ochsner students was significantly different from their domestic and international classmates. These differences suggest that the UQ-Ochsner students had a personality profile similar to that of their classmates but with levels of certain traits that in combination contribute to higher levels of resilience and a stronger sense of calling. These are desirable characteristics beneficial to coping in general. The findings suggest that this cohort of UQ-Ochsner students had a robust calling to the practice of medicine and were strongly resilient to overcome the challenges to achieve that goal. These findings may help inform aspects of recruitment into and counseling during the UQ-Ochsner medical program. This study invites a longitudinal follow-up study to investigate whether these differences result in more or less vulnerability to stress and burnout during medical school and into postgraduate training.

## Notes

*This article meets the Accreditation Council for Graduate Medical Education and the American Board of Medical Specialties Maintenance of Certification competencies for Interpersonal and Communication Skills and Professionalism.*
